# Endorsing a Hidden Plasmonic Mode for Enhancement of LSPR Sensing Performance in Evolved Metal–insulator Geometry Using an Unsupervised Machine Learning Algorithm

**DOI:** 10.1021/acsphyschemau.2c00033

**Published:** 2022-09-01

**Authors:** Nikhil Bhalla, Atul Thakur, Irina S. Edelman, Ruslan D. Ivantsov

**Affiliations:** † Nanotechnology and Integrated Bioengineering Centre (NIBEC), School of Engineering, Ulster University, Jordanstown, Shore Road, Newtownabbey, Northern Ireland BT37 0QB, United Kingdom; ‡ Healthcare Technology Hub, Ulster University, Jordanstown, Shore Road, Newtownabbey, Northern Ireland BT37 0QB, United Kingdom; § Amity Institute of Nanotechnology, Amity University Haryana, Gurugram, Haryana 122413, India; ∥ Kirensky Institute of Physics, FRC KSC Siberian Branch of Russian Academy of Sciences, Krasnoyarsk 660036, Russia

**Keywords:** LSPR, Plasmonics, PCA, Deconvolution, Sensors

## Abstract

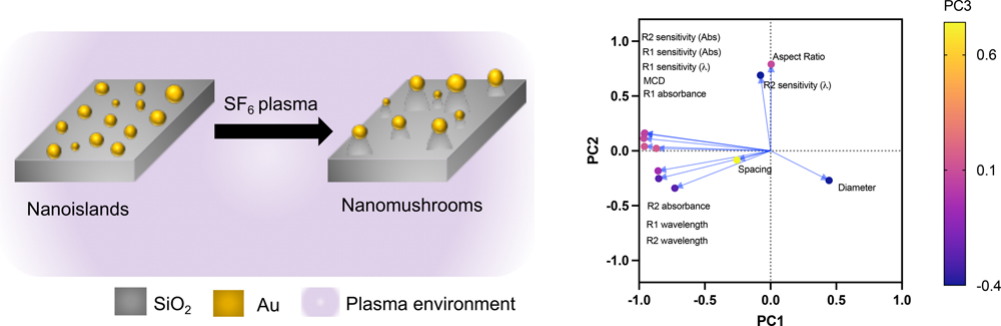

Large-area nanoplasmonic structures with pillared metal–insulator geometry, also called nanomushrooms (NM), consist of an active spherical-shaped plasmonic material such as gold as its cap and silicon dioxide as its stem. NM is a geometry which evolves from its precursor, nanoislands (NI) consisting of aforementioned spherical structures on flat silicon dioxide substrates, via selective physical or chemical etching of the silicon dioxide. The NM geometry is well-known to provide enhanced localized surface plasmon resonance (LSPR) sensitivity in biosensing applications as compared to NI. However, precise optical phenomenon behind this enhancement is unknown and often associated with the existence of electric fields in the large fraction of the spatial region between the pillars of NM, usually accessible by the biomolecules. Here, we uncover the association of LSPR enhancement in such geometries with a hidden plasmonic mode by conducting magneto-optics measurements and by deconvoluting the absorbance spectra obtained during the local refractive index change of the NM and NI geometries. By the virtue of principal component analysis, an unsupervised machine learning technique, we observe an explicit relationship between the deconvoluted modes of LSPR, the differential absorption of left and right circular polarized light, and the refractive index sensitivity of the LSPR sensor. Our findings may lead to the development of new approaches to extract unknown properties of plasmonic materials or establish new fundamental relationships between less understood photonic properties of nanomaterials.

## Introduction

Refractive index sensitive localized surface plasmon resonance (LSPR) sensors have been widely used for bio/chemical sensing applications.^1^ Essentially, arrays of noble metal nanostructures, of sizes less than the absorption wavelength of a given metal, are commonly printed on glass or silicon substrates to develop LSPR sensors.^2^ The LSPR spectrum of these nanostructures, recorded in transmission or reflection mode, is sensitive to the changes in the refractive index (RI) of the surrounding medium.^3^ This forms the crux of the bio/chemical sensing where a variety of chemicals in solution form or biomolecules can be attached on the nanostructure surface, yielding shifts in the wavelength/absorbance of the LSPR spectrum.^4^ These wavelength/absorbance shifts are then correlated to the concentration of the attached bio/chemical entity to develop the LSPR sensor.^5^ The sensitivity of the LSPR sensor is based on several factors, such as size, shape, and aspect ratio of the nanostructure, inter-nanostructure distance, and dielectric constant of the material.^6^

Among the aforementioned factors, engineering the shape/geometry of the nanostructure is one of the common approaches used to enhance the RI sensitivity of the LSPR sensor as it ensures that the material related properties, mostly chemical properties, of the chosen material are utilized within the sensor system.^7^ For instance, the array of gold nanoparticles deposited on the glass surfaces, often referred as nanoislands (NI), is used as a robust platform for a range of bio/chemical sensing applications both in academia and in industry.^8−10^ The major chemical advantage of the material gold over other materials is to provide antioxidation properties^11^ to the surface while the spherical shape is easy to fabricate in large arrays, and which is also reported to aid in enhanced immobilization of the biomolecules.^12^ However, despite the advantages, the spherical shaped nanostructures are less sensitive to changes in the refractive index as compared to some other shapes such as nanoscale stars,^13^ pyramids,^14^ spikes,^15^ rods,^16^ and many others.^17^ Amidst these shapes, one easy to develop new geometry from NI, by following simple post processing of the large-area NI, is mushroom shaped nanostructures, hereafter called nanomushrooms (NM).^18^ The NM structure is developed by lifting metal nanostructures above the substrates with pillars achieved by either selective chemical or physical etching of the substrate.^19^ It is reported that the RI sensitivity of the resultant LSPR sensors is increased in such structures because a large fraction of the spatial region with enhanced electric fields is exposed to the environment and is accessible by the biomolecules.^20^

Most often, the absorbance peak of the large area NI structures has a wide full width half-maximum (fwhm) which can be deconvoluted mathematically.^21^ The process of deconvolution decomposes the original wide absorbance spectrum into multiple peaks which can be associated with different modes of the absorbance present in a given material. In this work, we have deconvoluted the absorbance peaks and studied the relationship of the different modes (LSPR modes) with the change in the refractive index of NI and NM based geometries. Additionally, with the help of magnetic circular dichroism (MCD), we provide information about the intrinsic properties of the NI and NM which are correlated to the LSPR modes, type of nanostructure geometry, and refractive index changes using principal component analysis (PCA). Note that most reports in the literature investigated MCD for ensembles of gold nanoparticles dispersed in liquid or gels where the influence of the media on the MCD was inevitable.^22,23^ Here, we are dealing with nanostructures of the same size and shape fixed uniformly on a solid surface and thus have boundaries only with this solid surface or air. Furthermore, the use of PCA as an unsupervised machine learning algorithm has several applications ranging from exploratory data analysis, dimensionality reduction, information compression, and data denoising. Additionally, PCA is based on linear algebra, which is computationally easy to solve by computers. Therefore, use of PCA in this work allows high reliability for correlating various absorbance modes in an artificially intelligent manner.

## Results and Discussion

**Figure 1:**
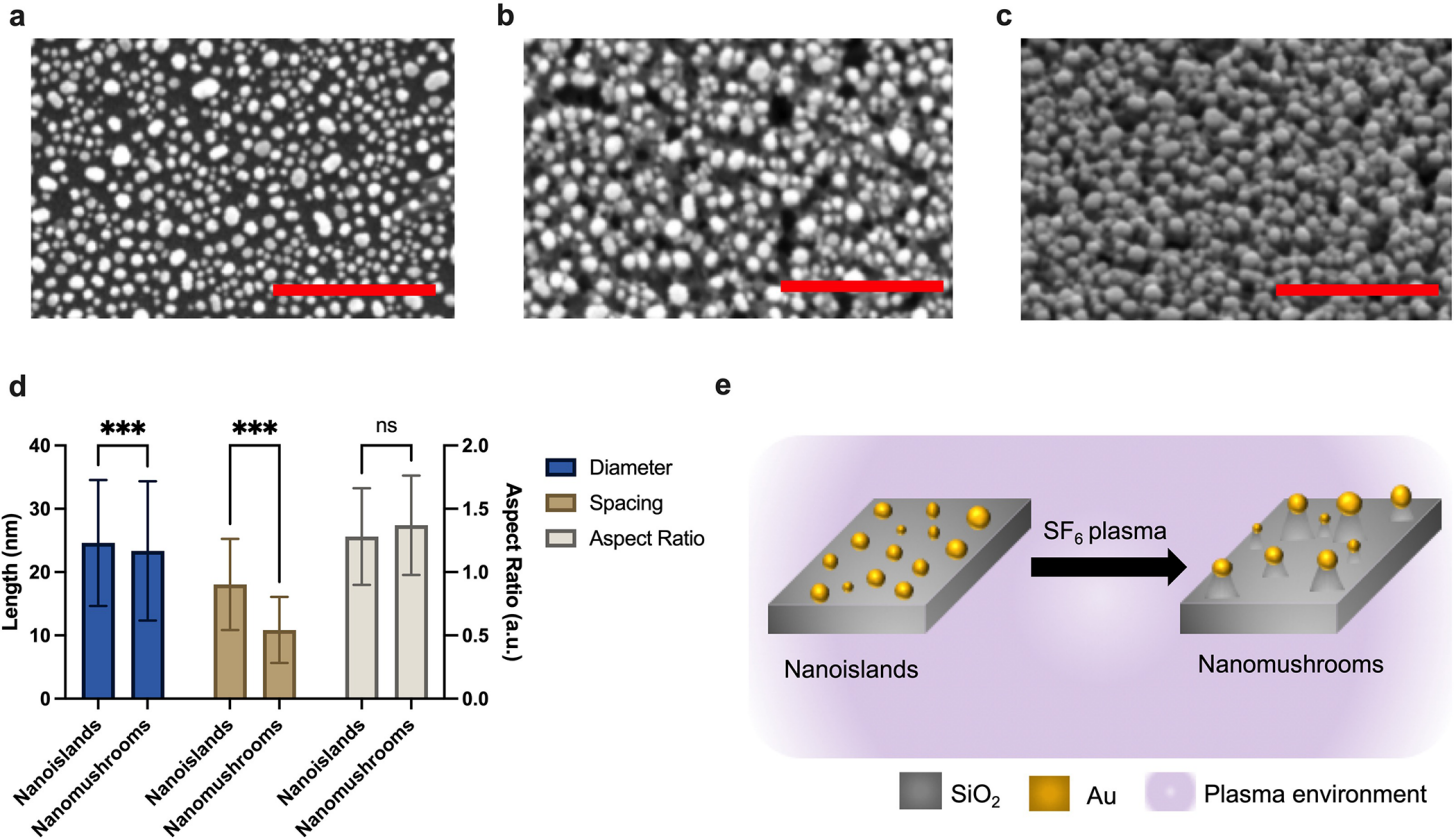
Morphological characterization of nanostructures: (a) top view of nanoisland (NI) and (b) top view of nanomushroom (NM) type nanostructures on glass substrates; (c) 45° titled substrate consisting NM type nanostrutcures. Note that in panels (a)–(c) the scanning electron microscopy images were obtained at 100,000× magnification. The length of the red scale bar in all images is 400 nm. (d) Comparison of mean diameter, inter-nanostructure spacing, and aspect ratio of the NI and NM; (e) schematic of process showing NM generation from NI via reactive ion etching. The asterisks (*) in panel (d) indicate the level of significance and “ns” stands for “not significant” in the Šídák multiple comparison test.

Figure 1 shows the morphological features of the developed NI and NM type LSPR sensors. Essentially, the top view of the NI and NM substrate is shown in Figure 1a and b, respectively. The side view of the NM substrate, showing the stems of the NM, can be observed in Figure 1c. The statistical data of the diameter, inter-nanostructure spacing (called spacing), and aspect ratio of the gold caps, i.e., the ratio of its major to minor axis, are plotted in Figure 1d. Additionally, we also show the schematic of the process which allows NI geometry to evolve into NM geometry in Figure 1e. The mean diameter of NI was 24.60 nm as compared to 23.34 nm of NM. The mean diameter of the NM decreases as some of the Au of NI is etched and falls back on the glass substrate, as also seen our previous work.^18^ The decrease in the average spacing between nanostructures in the NM substrates (10.86 nm) as compared to the NI substrates (18.05 nm) further suggests that some gold which is etched off in the reactive ion plasma and falls back on the surface. The mean aspect ratio of the NM also increases from 1.28 in NI to 1.37.

**Figure 2:**
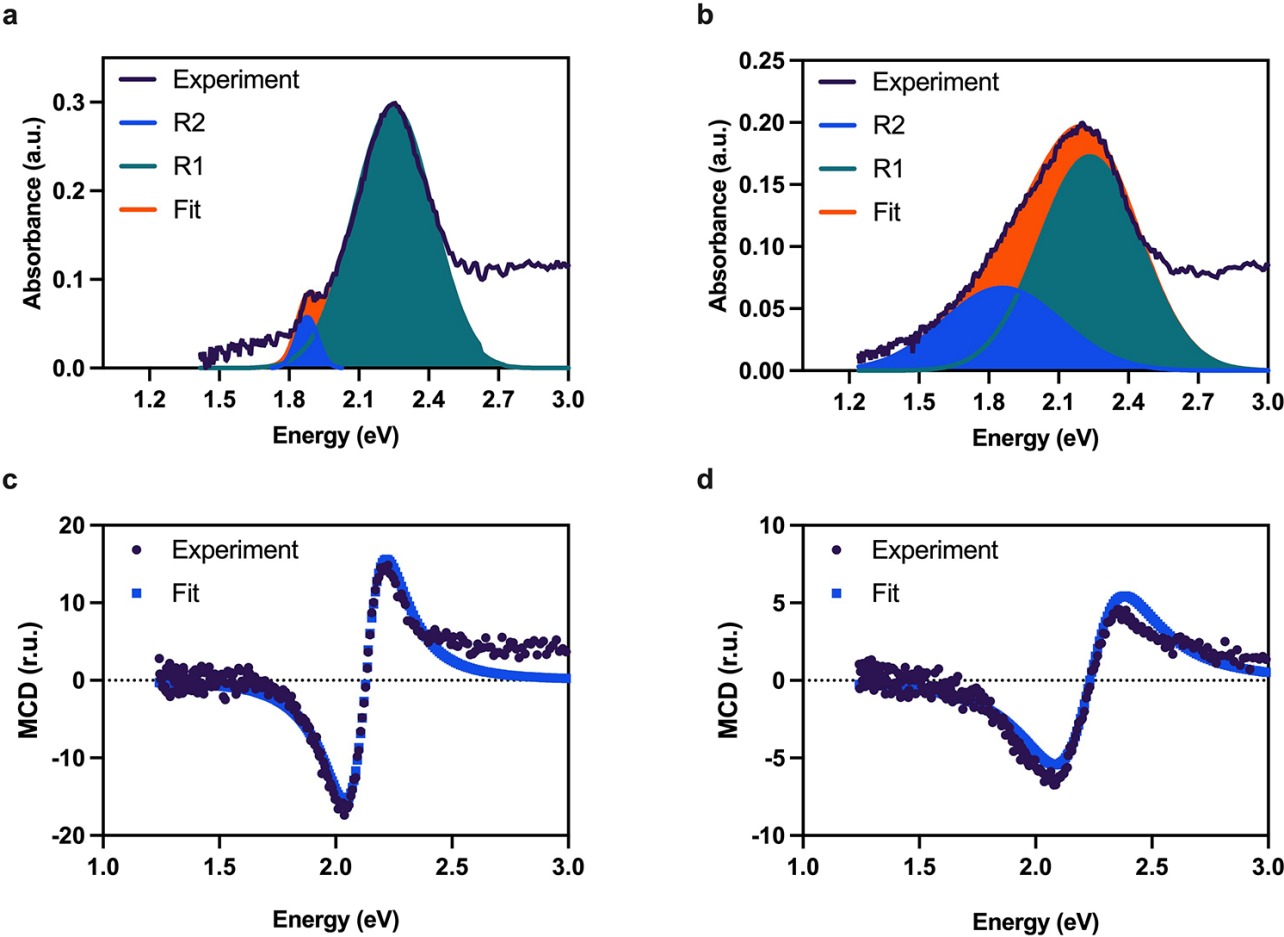
Ultraviolet–visible spectroscopy and magnetic circular dichroism (MCD): (a) absorbance spectra of nanoislands (NI) and (b) absorbance spectra of nanomushrooms (NM). Both panels (a) and (b) consist of deconvoluted peaks corresponding to two localized surface plasmon resonance modes, R1 and R2, of NI and NM along with the superimposed fit of the deconvoluted peaks. (c) MCD of NI and its Lorentz counter and (d) MCD of NM and its Lorentz counter. Note all spectra are obtained at 25 °C in a magnetic field of 1.3 T.

The absorbance of NI and NM pristine samples as measured in air are shown in Figure 2a and b. The MCD spectra of NI and NM samples are shown in Figure 2c and d. Note that both MCD and absorption spectra are measured at room temperature of 25 °C.

MCD is determined as a difference between absorption coefficients (*Δk*) for light waves right (k_+_) and left (k_–_) circularly polarized relatively to the direction of the magnetic vector of the matter placed in a magnetic field. In this classical case, MCD is described by eq 1.
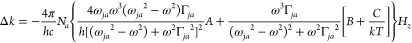
1Here *N*_*a*_ is the number of absorption centers in the *a* state per unit volume, ω is the current frequency, and Γ_*ja*_ and ω_*ja*_ are the half-width and the frequency, respectively, of the spectral line corresponding to the transition from the *a* to *j* state. Three contributions *A*, *B*, and *C* in eq 1 describe three different types of electron transitions.^24^ In brief, the diamagnetic term *A* originates from the electronic states splitting in magnetic field *H.* Note that *A* is proportional to the *H* value and does not depend on the temperature and this term becomes zero at ω = ω_*ja*_. The paramagnetic term *C* is due to the difference between the thermal occupation of the components of the ground state split by *H*, and hence, it is proportional to *H* and depends on the temperature. The term *B* is determined by the mixing of the states in the applied magnetic field, and it is independent of temperature and has the same spectral dependence as the *C* term. Past reports^25,26^ have considered features of the MCD spectrum formation in the region of plasmon resonance in nanoparticles of nonmagnetic noble metals taking into account an action of the Lorentz force on the free electrons motion in the presence of a magnetic field. This force lifts the degeneracy of plasmon oscillations excited by right and left circularly polarized (RCP and LCP) light waves. Thus, the resonant plasmon frequencies become equally shifted toward the red or the blue for RCP or LCP waves relative to the frequency of the degenerated plasmon excitation. As MCD is a difference between the RCP and LCP wave absorptions, its spectrum takes the form of an S-shaped line which is characteristic of the diamagnetic *A* term described by the first term in eq 1. The MCD spectra studied for gold nanoparticle solutions elsewhere^22,26^ demonstrated an S-shaped line passing through zero at the energy of the light wave corresponding to the maxima of the LSPR absorbance peak in full accordance with the theoretical consideration. In these reports, the observed S-shaped line was asymmetric; i.e., the higher energy positive peak was of lower intensity compared to the lower energy negative one. This asymmetry was explained with the interband electron transition in gold nanoparticles around 2.38 eV, which was closer to the positive peak, and therefore the intensity of the positive peak changes more noticeably.

In contrast to the solution based plasmonic samples, in our study, the active plasmonic material is present in the solid state on the glass substrates. The absorption spectra of both NI and NM in Figure 2a and b are typical of the LSPR and well fitted to two Lorentz counters with energy values of their gravity centers of E1 = 2.23 eV and E2 = 1.86 eV in both cases. Thus, one can say that two LSPR modes contribute to the observed absorption line: R1 and R2. In the case of NI, the R2 intensity is more than 10 times less compared to the R1 intensity. In the case of mushrooms, the R2 intensity is about half the intensity of the R1 intensity; i.e., it increases strongly relative to the NI case. Such a change can be associated with the transition from the island geometry to the mushroom geometry. Essentially, the NM consist of two parts, an oval cap and an oblong stem, which makes it possible to compare these results with those reported by Han et al.,^27^ in which the absorption and MCD spectra were recorded for spherical gold nanoparticles and nanorods with a cross-sectional diameter that is the same as that of their chosen spherical nanoparticles. The LSPR energies of 2.37 and 1.8 eV were reported for spherical nanoparticles and nanorods, respectively.^27^ The closeness of these energy values of nanoparticles and nanorods to the R1 and R2 resonances in our samples allows us to correlate R1 and R2 with LSPR in the mushroom caps and stems, respectively.

The MCD spectrum of NI in Figure 2c demonstrates the ideal diamagnetic shape similar to the MCD spectrum of spherical gold nanoparticles in solution, reported in the literature. It fits well to the Lorentz curve with the equal intensities of positive and negative peaks and also coincides in shape with the MCD spectrum of gold nanodisk antennas on glass substrates reported in the literature.^26^ However, for the NM sample, the MCD spectrum deviates from the correct shape and noticeably broadens, which can be attributed to the superposition of LSPR modes R1 and R2, where the R1 intensity is strong due to the presence of mushroom caps and stems. A similar MCD spectrum was also observed for nanorods by Han et al.,^27^ with the intense negative peak near 1.8 eV and nonsymmetric S-shape contribution at higher energies (positive peak). Note that the cross-sectional part of the nanorods can be considered similar in geometry to NM cap and the length can geometrically be compared with the stem of the NM. Therefore, by analogy, we can say that the negative peak of the MCD is attributed to the NM stem which leads to broadening of the overall absorbance including similarity in additional features of the overall MCD response at higher energies.

**Figure 3:**
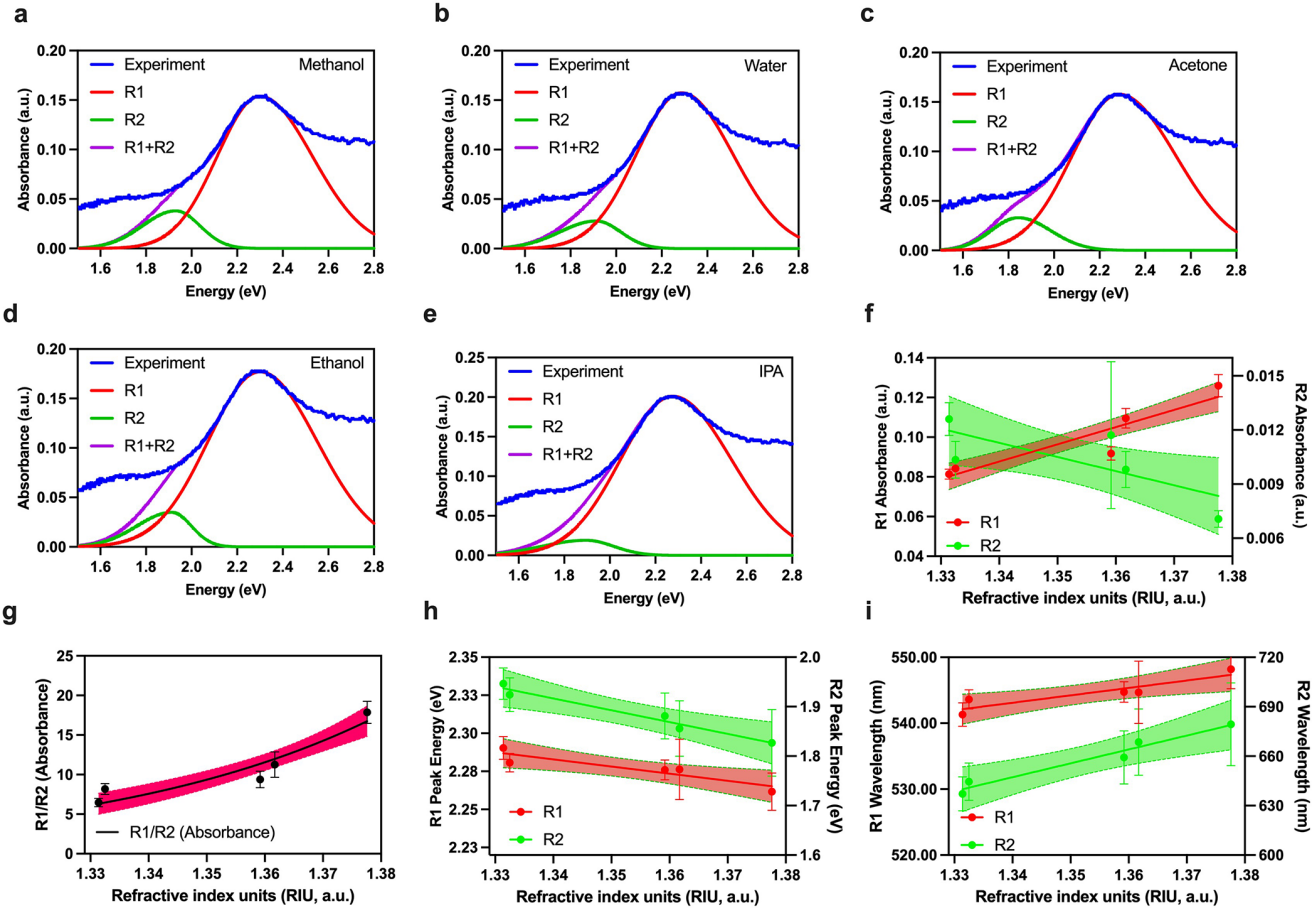
Refractive index characterization of nanoislands (NI): absorbance spectra of NI upon exposure to (a) methanol, (b) water, (c) acetone, (d) ethanol, and (e) isopropanol. Panels (a)–(e) consist of deconvoluted peaks corresponding to two localized surface plasmon resonance (LSPR) modes, R1 and R2, along with the superimposed fit of the deconvoluted peaks. (f) Absorbance shifts upon refractive index change of NI; (g) ratio of absorbance recorded from R1 and R2 modes of the LSPR from NI plotted vs change in the refractive index. Panel (h) shows how peak energy shifts with changes in the refractive index of NI, and panel (i) depicts changes in the wavelength upon changes in the refractive index of NI. Note that the shaded region in (f)–(i) represent the 95% confidence interval of the respective fit. Panels (f), (h), and (i) show a linear fit, and panel (g) shows an exponential fit. Within panels (f)–(i), each point consist of at least 6 replicates, *n* = 6.

We extended our finding to investigate the impact of the refractive index (RI) change on the R1 and R2 modes of the LSPR substrate. It is well-known experimentally that the NM substrate yields higher RI sensitivity, as also shown in our past work on refractive index biosensing using NM substrates.^18,28^ In Figure 3, we show the refractive index response of the NI substrate in water (RI: 1.3325) and harsh organic solvents including methanol (RI: 1.3314), acetone (RI: 1.3592), ethanol (RI: 1.3617), and isopropanol (RI: 1.3776); see Figure 3a–e.

These absorbance spectra are deconvoluted into the aforementioned R1 and R2 modes between 1.6 and 2.8 eV, and absorbance (total area under the curve) and peak shifts are plotted in Figure 3f–i. Note that the R2 mode broadens in a liquid environment as compared to the pristine NI sample. This anomalous broadening of the R2 peak can be associated with a minute amount of gold leaching in the liquid environment, leading to a decrease in the particle size for which broadening of the LSPR is previously observed in the literature.^23^ The changes in the absorbance shown in Figure 3f depict a linear increase in the absorbance of R1 and a linear decrease in the absorbance of R2. However, the absorbance ratio of R1/R2 increases (Figure 3g) exponentially, suggesting that the absorbance of R2 is less dominant than R1 in contributing toward the total absorbance change due to the change in the refractive index. The energy peak shifts and its translation to wavelength shifts are plotted in Figure 3h and i, respectively. We observe a red shift in the wavelength upon change in the refractive index, which is a typical response of the LSPR sensing to refractive index change. The mean sensitivity of the R1 and R2 modes are deduced from the slope of the plots in Figure 3h and i, which are found to be 112.5 and 842.1 nm/RIU for R1 and R2, respectively.

**Figure 4:**
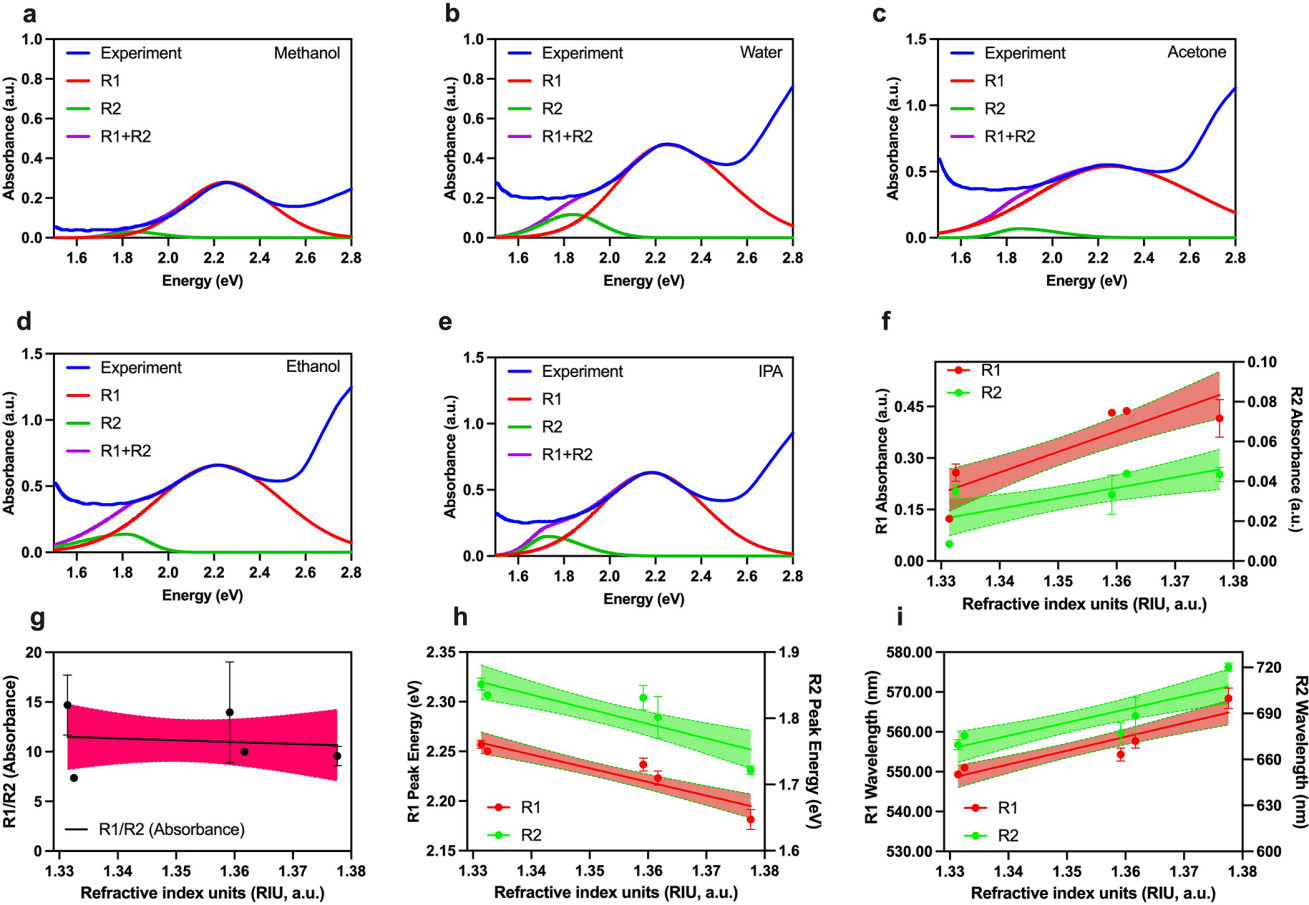
Refractive index characterization of nanomushrooms (NM): absorbance spectra of NM upon exposure to (a) methanol, (b) water, (c) acetone, (d) ethanol, and (e) isopropanol. Panels (a)–(e) consist of deconvoluted peaks corresponding to two localized surface plasmon resonance (LSPR) modes, R1 and R2, along with the superimposed fit of the deconvoluted peaks. (f) Absorbance shifts upon refractive index change of NM; (g) ratio of absorbance recorded from R1 and R2 modes of the LSPR from NM plotted vs change in the refractive index; panel (h) shows how peak energy shift with change in the refractive index of NM, and panel (i) depicts changes in the wavelength upon change in the refractive index of NM. Note that the shaded region in panels (f)–(i) represent the 95% confidence interval of the respective fit. Panels (f)–(i) show a linear fit, and each point consist of at least 6 replicates, *n* = 6.

Similarly, we characterized the NM substrate for RI sensitivity. The deconvoluted R1 and R2 modes of the LSPR response of the NM are plotted in Figure 4a–e for the aforementioned chemical solutions. The associated absorbance changes of the R1 and R2 modes, which both show a linear increase in absorbance when refractive index is changed, are plotted in Figure 3f. The ratio of R1/R2 (Figure 4g), however, remains constant in comparison to NI, earlier discussed in Figure 3g, as suggested by the shaded region of the linear fit corresponding to the 95% confidence interval of the fit. The peak shifts in terms of energy and wavelength are shown in Figure 4h and i. In comparison to NI, a higher mean sensitivity of R1 and R2 modes is found in NM: 346.3 and 855.8 nm/RIU.

**Figure 5:**
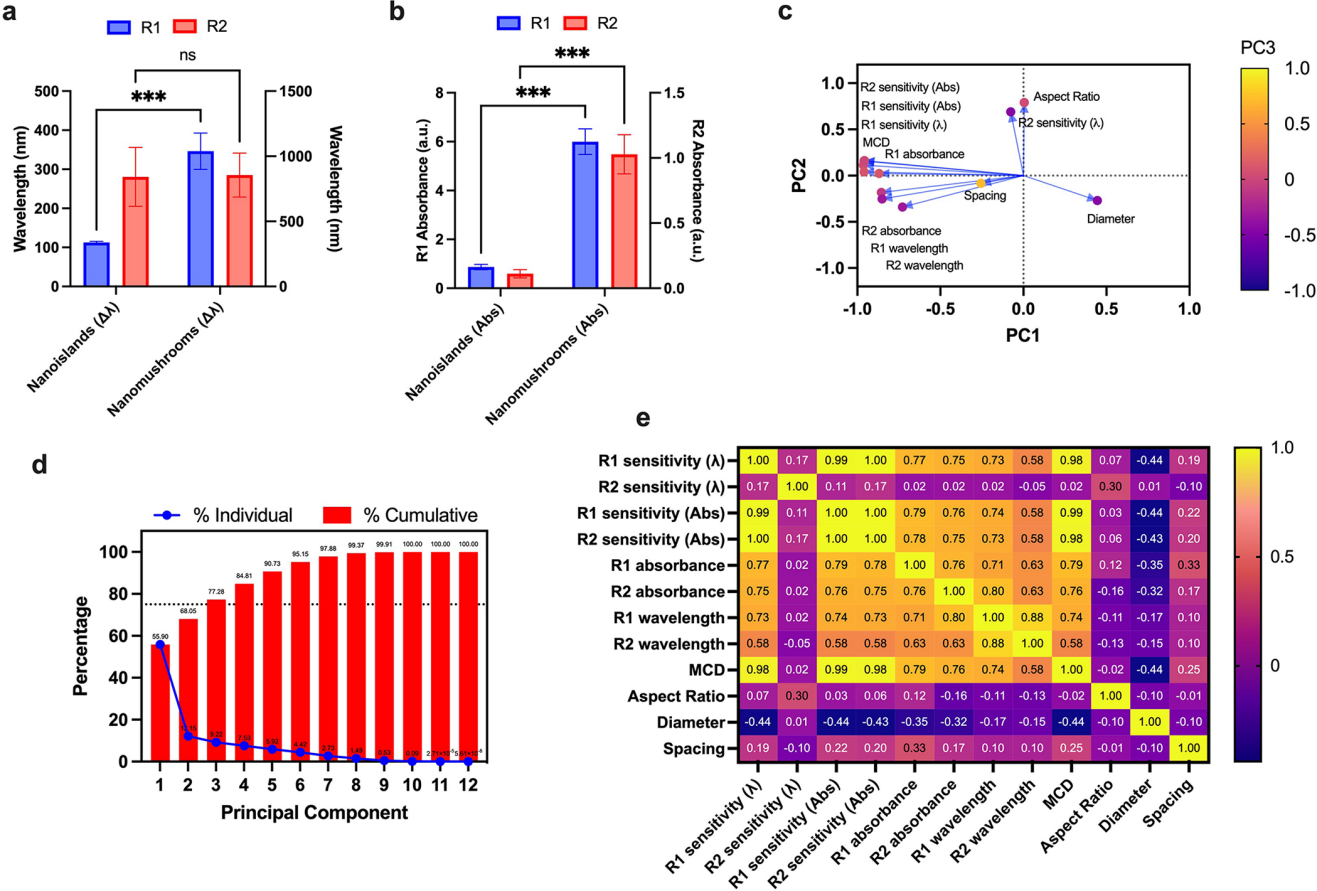
Statistical analysis: (a) comparison of NI and NM refractive index sensitivity for change in wavelength for R1 and R2 modes of localized surface plasmon resonance (LSPR); (b) comparison of NI and NM refractive index sensitivity for change in absorbance for R1 and R2 modes of LSPR; (c) principal component analysis (PCA) showing relationship between 3 principal components (PA) out of 12 variables studied for the PCA; (d) percentage of variance in the 12 PC. The dotted line represents the 75% cutoff chosen to select the PC. Note that PC1, PC2, and PC3 have a cumulative variance of 77.28% which are selected for the analysis; (e) correlation matrix showing Pearson’s correlation between the 12 variables. The askterisks (*) in panels (a) and (b) indicate the level of significance and “ns” stands for “not significant” in the Šídák multiple comparisons test.

We compared the sensitivities in more detail using Šídák’s multiple comparisons test, Figure 5a and b, which shows statistically significant differences between the refractive index sensitives of only R1 modes of NI and NM. Note that the R2 mode did not show any significant differences in the sensitivity of wavelength shifts; see Figure 5a. Therefore, the wavelength shift enhancement observed in NM geometry upon changes in the refractive index can be attributed to the R1 LSPR mode. It is also noteworthy to see that the refractive index sensitivity of absorbance shifts is significantly higher for both R1 and R2 modes in NM compared to NI; see Figure 5b. This data corresponds to the slopes of absorbance measurements in Figures 3f and 4f. To further validate our observation of the R1 mode contributing more to the change in the refractive index, we performed principal component analysis (PCA) of all parameters obtained from the LSPR measurement and the structural features (diameter, spacing, and aspect ratio) of the NI and NM substrates; see Figure 5c–e. As mentioned earlier, PCA is a type of unsupervised machine learning technique which can be used for finding relationships between different variables. We selected 3 principal components (PC) out of the 12 variables (all 12 variables are shown in Figure 5e). The method of selection ensures the total amount of variance in the data set, at least 75%, which the components represent collectively; see more details in Methods. Essentially, we have selected PC1, PC2, and PC3 which represent 77.28% of the total variance in the data set; see Figure 5d. The relation between these 3 PC is demonstrated in Figure 5c which shows that the wavelength dependent sensitivity of the R2 mode is affected by the aspect ratio of the NI and NM geometry, as these points are close to each other in the biplot showing the PC relationships. Further evidence for this is provided in Figure 5d, which shows that while the overall wavelength dependent sensitivity of the material is generally dependent on the R1 mode, the R2 wavelength dependent sensitivity is mostly affected by the aspect ratio of the nanostructure (0.30 value of correlation of the R2 wavelength sensitivity and aspect ratio, the highest value for R2 wavelength sensitivity in the matrix). It is also interesting to note that the R2 and R1 wavelengths and R2 absorbance have a strong correlation among them while R1 absorbance is independent of these 3 parameters. This can be attributed to the fact that the spacing of the nanostructures mostly affects the R1 absorbance as compared to its effect on the R2 and R1 wavelengths and R2 absorbance; therefore, we can say that spacing is strongly correlated to the changes in R1 absorbance. Additionally, the MCD absorbance is also observed to vary more with R1 absorbance, suggesting that the spacing of the nanostructures affects the differential absorbance of the overall substrate. Similar nanostructure spacing associated changes in the MCD are reported elsewhere for magnetic particles.^29^

## Conclusion

In summary, we find that different modes of the LSPR affect the refractive index sensitivity of the sensor. These modes are sensitive to the changes in the geometry of the nanostructure, thereby contributing to the differences in the sensitivity of the sensor from one geometry to the other. The extension of our work could lead to mode specific LSPR bio/chemical sensing using nanostructures where each mode can be associated with a specific response of the sensor.^30^ These will improve the translation of a diverse range of biosensing systems involving live cells and nucleic acid and amino acid based molecules.^28,31,32^ With the advent of new machine learning algorithms and artificial intelligence, different modes of absorption can be made easy to understand and interpret by the user.^33^ The recent discovery of the plasmonics and magnetic effects in doped semiconductors has opened up the field to new sensing materials,^34,35^ where doping can lead to different plasmonic modes that can be studied on a similar basis and correlation between unknown physical parameters or sensing performance can be understood as shown by this work. Therefore, the developed approach of using machine learning algorithms with deconvolution may lead to the discovery of new fundamentals or allow the exploration of material properties beyond the current state of the art in characterization and analysis of plasmonic materials.

## Methods

### Sensor Fabrication

The NI based LSPR sensor was fabricated by a two-step process. First, we deposited 5 nm Au film onto a SiO_2_ substrate at 0.1–0.2 Å s^–1^ using an e-beam evaporator (KE604TT1-TKF1, Kawasaki Science) in a class 1000 clean room. The substrates were cleaned with acetone and isopropanol prior to deposition. Next we annealed the 5 nm gold film at 560 °C for 3 h to generate a distribution of NI on the SiO_2_ substrate. To create NM, we selectively etched the SiO_2_ on the NI sensor to generate mushroom-like structures using reactive ions of SF_6_. The reactive ion etching (RIE) was performed for 5 min by using inductively coupled plasma (ICP) chemical vapor deposition equipment (Plasmalab 100, Oxford Instruments) at a pressure of 10 mTorr and a flow rate of 45 sccm (standard cubic centimeters per minute) SF_6_. The RF power coil and the RF bias coils were fixed to 150 and 10 W, respectively, and the temperature inside the plasma chamber was maintained at 5 °C. More details on the fabrication techniques can be found in our past work.^18,28^

### LSPR Measurement Setup

The LSPR signal was acquired using a homemade, in Ulster University-UK, setup which consists of components purchased from Ocean Insight: spectrometer FLAME-T-XR1-ES, UV–vis patch connectors, DH-2000 Deuterium-Tungsten Halogen lamp (DH 2000-S-DUV-TTL), RTL-T stage, and Ocean View software. Prior to the acquisition of the LSPR spectrum, dark and reference signals for background noise cancellation were measured using a glass slide as a reference. This glass slide was the same substrate on which NI and NM were deposited. All generated data was analyzed and plotted using the built-in functionality of the GraphPad Prism 9 software. Note that some optical absorption spectra were recorded with the use of a spectral ellipsometer, designed and manufactured at the Rzhanov Institute of Semiconductor Physics of the Siberian Branch of the RAS (Novosibirsk, Russia) to cross-check the absorbance measurements conducted at Ulster University-UK.

### Scanning Electron Microscopy (SEM)

A small section of the developed NI or NM substrate was cut from the original sensor using a diamond-tipped glass cutter and attached to a scanning electron microscope, FEI Quanta 250 FEG mount using carbon tapes. SEM measurements were taken at 5 eV to obtain high-resolution images with magnification of 100k×. Prior to imaging, NI and NM were coated with palladium–platinum (Pd–Pt) using ion sputtering (Ion Sputter MC1000) to avoid sample charging.

### MCD

MCD was measured in the 1.2–3.5 eV energy interval using a homemade spectropolarimeter setup designed at the Kirensky Institute of Physics based on the MDR-2 monochromator. Modulation of the light wave polarization from right to left circular polarization relative to the magnetic field direction was used. The modulator was made of a fused quartz prism with an attached piezoceramic element. A standing elastic wave propagating along the horizontal axis of the prism was excited in it by applying an ac electric signal to a piezo element with a frequency ω corresponding to the eigenfrequency of the system which led to the appearance of optical anisotropy in the prism. When the compression acoustic half-wave propagates through the prism, its horizontal axis becomes the “slow” axis of the prism. And when the second stretching half-wave passes through it, the horizontal axis becomes the “fast” axis of the prism. If a linearly polarized light wave with a plane of polarization making an angle of 45° with respect to this axis is incident on a prism perpendicular to its long axis, then at the exit from the prism the wave will have circular polarization changing from right to left with a frequency ω. Passing this through a sample with MCD, that is, having different absorption coefficients for right and left polarized waves, the light becomes modulated in intensity where the modulation amplitude is proportional to the MCD signal value. The MCD measurements were performed at an applied magnetic field equal to 1.3 T at 25 °C. The sensitivity of the MCD measurements was 10^–5^, and the spectral resolution was 20 cm^–1^.

### PCA

Principal component analysis (PCA) was conducted using a built-in multiple variable analysis tool with GraphPad Prism 9. We used a classical method based on the Kaiser–Guttman rule (also known as the Kaiser criterion) to select PC. Here each of the principal components (linear combination/mixture of starting variables) is uncorrelated and the information from the initial variables is compressed into these via orthogonal eigenvectors that represent the maximal amount of variance in the data set. Thus, the PCA essentially extracts the smallest number of components that describe the most variation of the original data set with minimal loss of information. In this method, we assume that, with the standardized data, the variance of each of the original variables is equal to 1. Therefore, a PC with an eigenvalue greater than 1 contains more variance than a single variable in the original data. Additionally, we have also checked the percentage of total variance explained by each of the principal components and ensured that the selected components have at least 75% of the total variance in our original data. The statistical analysis is based on the Pearson correlation linear regression fit which computes the correlation (goodness of fit with *r*^2^) between all variables in the form of a correlation matrix. A two-tailed test with a confidence interval of 99% is used to compute the *P*-value. In this test, a value of 1 or 0 represents a perfect or no correlation, respectively, values between 0 and 1 represent a concurrent increase or decrease of two variables, and finally values from −1 to 0 shows an inverse relationship between the two variables.
